# Insight into the Mechanisms of Carbapenem Resistance in *Klebsiella pneumoniae*: A Study on IS26 Integrons, Beta-Lactamases, Porin Modifications, and Plasmidome Analysis

**DOI:** 10.3390/antibiotics12040749

**Published:** 2023-04-13

**Authors:** Chien-Hao Tseng, Yao-Ting Huang, Yan-Chiao Mao, Chung-Hsu Lai, Ting-Kuang Yeh, Chung-Mei Ho, Po-Yu Liu

**Affiliations:** 1 Division of Infectious Diseases, Department of Internal Medicine, Taichung Veterans General Hospital, Taichung 40705, Taiwan; 2 Department of Computer Science and Information Engineering, National Chung Cheng University, Chia-Yi 62102, Taiwan; 3 Division of Clinical Toxicology, Department of Emergency Medicine, Taichung Veterans General Hospital, Taichung 40705, Taiwan; 4 School of Medicine, National Defense Medical Center, Taipei 11490, Taiwan; 5 Department of Post-Baccalaureate Medicine, College of Medicine, National Chung Hsing University, Taichung 402, Taiwan; 6 Division of Infectious Diseases, Department of Internal Medicine, E-Da Hospital, Kaohsiung 82445, Taiwan; 7 School of Medicine, College of Medicine, I-Shou University, Kaohsiung 82445, Taiwan; 8 Genomic Center for Infectious Diseases, Taichung Veterans General Hospital, Taichung 40705, Taiwan; 9 Rong Hsing Research Center for Translational Medicine, National Chung Hsing University, Taichung 402, Taiwan; 10 Ph.D. Program in Translational Medicine, National Chung Hsing University, Taichung 402, Taiwan

**Keywords:** antimicrobial resistance determinants, plasmid-mediated resistance, plastidome analysis, carbapenem-resistant *Klebsiella pneumoniae*

## Abstract

The emergence of carbapenem-resistant *Klebsiella pneumoniae* poses a significant threat to public health. In this study, we aimed to investigate the distribution and genetic diversity of plasmids carrying beta-lactamase resistance determinants in a collection of carbapenem-resistant *K. pneumoniae* blood isolates. Blood isolates of carbapenem-resistant *K. pneumoniae* bacteremia were collected and identified. Whole-genome sequencing, assembly and analysis were performed for the prediction of antimicrobial resistance determinants. Plasmidome analysis was also performed. Our plasmidome analysis revealed two major plasmid groups, IncFII/IncR and IncC, as key players in the dissemination of carbapenem resistance among carbapenem-resistant *K. pneumoniae*. Notably, plasmids within the same group exhibited conservation of encapsulated genes, suggesting that these plasmid groups may serve as conservative carriers of carbapenem-resistant determinants. Additionally, we investigated the evolution and expansion of IS26 integrons in carbapenem-resistant *K. pneumoniae* isolates using long-read sequencing. Our findings revealed the evolution and expansion of IS26 structure, which may have contributed to the development of carbapenem resistance in these strains. Our findings indicate that IncC group plasmids are associated with the endemic occurrence of carbapenem-resistant *K. pneumoniae*, highlighting the need for targeted interventions to control its spread. Although our study focuses on the endemic presence of carbapenem-resistant *K. pneumoniae*, it is important to note that carbapenem-resistant *K. pneumoniae* is indeed a global problem, with cases reported in multiple regions worldwide. Further research is necessary to better understand the factors driving the worldwide dissemination of carbapenem-resistant *K. pneumoniae* and to develop effective strategies for its prevention and control.

## 1. Introduction

Antibiotic resistance is a growing public health threat that affects millions of people worldwide [1]. *Klebsiella pneumoniae*, a common cause of healthcare-associated infections, has become a major concern due to its increasing prevalence of carbapenem resistance [2]. Carbapenem-resistant *K. pneumoniae* (CRKP) infections are associated with high morbidity and mortality rates, making them a significant burden on healthcare systems [3].

The development and spread of carbapenem resistance in *K. pneumoniae* are driven by a combination of factors, including the use of antibiotics, horizontal gene transfer, and selective pressure in clinical settings [4,5]. In particular, the acquisition of β-lactamases, such as extended-spectrum β-lactamases (ESBLs) and carbapenemases, is a significant driver of antibiotic resistance in *K. pneumoniae* [6,7].

Extended-Spectrum Beta-Lactamases (ESBLs) are enzymes produced by some bacteria that can break down and inactivate a wide range of beta-lactam antibiotics, including penicillins, cephalosporins, and monobactams [8]. Bacteria-producing ESBLs are often resistant to multiple antibiotics, which makes it difficult to treat infections caused by these organisms [9].

Carbapenem-resistant (CR) organisms are bacteria that have developed resistance to carbapenems, a class of broad-spectrum beta-lactam antibiotics often used as a last resort to treat severe infections caused by multidrug-resistant Gram-negative bacteria [10]. Carbapenem resistance typically occurs through the production of carbapenemase enzymes, which hydrolyze and inactivate carbapenems, or through alterations in outer membrane proteins and efflux pumps that reduce drug uptake and increase drug efflux [11].

In this study, we investigate the distribution of carbapenem-resistant determinants in *K. pneumoniae* strains, with a focus on the diversity of β-lactamases and the role of plasmids in the dissemination of resistance determinants. We also explore the potential role of IS26 integrons in the evolution and expansion of carbapenem resistance in these strains.

## 2. Results

### 2.1. Distribution of Carbapenem-Resistant Determinants in K. pneumoniae

**Figure 1:**
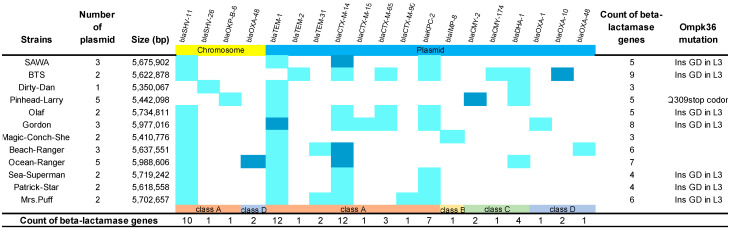
Distribution of carbapenem-resistant determinants in *Klebsiella pneumoniae* strains. This heat map illustrates the distribution of β-lactamase genes, their copy numbers, and OmpK36 mutation among *K. pneumoniae* strains from our study. Each row represents a single strain, and each column represents a specific β-lactamase gene. The color scale ranges from light blue, indicating a single copy of the gene, to dark blue, indicating two copies of the gene. The β-lactamase genes are divided into four classes (A to D) based on their amino acid sequence homology and functional properties. Class A, C, and D enzymes are serine-based, while class B enzymes are metallo-β-lactamases [12].

In this study, 12 carbapenem-resistant *K. pneumoniae* isolates were sequenced using third-generation sequencing techniques. Sequencing typing and antimicrobial susceptibility results are presented in Supplementary Table S1. The isolates were assembled into circular chromosomes and plasmids, with the number of plasmids ranging from 1 to 5 (Supplementary Table S2). A resistome analysis was performed to identify the presence and diversity of β-lactamases (classes A–D) in the isolates. The results revealed that 14 β-lactamases were located on chromosomes and 51 were located on plasmids (Figure 1, Supplementary Table S3). All of the strains encoded more than three β-lactamase genes, with the number ranging between three to nine. Class A β-lactamases were the most prevalent, accounting for 86% (12/14) of the chromosomal β-lactamases and 76% (39/51) of the plasmid-borne β-lactamases. 

The study revealed that ESBLs were the most commonly identified β-lactamases among the CRKP isolates. All of the most commonly found chromosomal β-lactamases, *bla*_SHV-11_ (83%, 10/12), were ESBLs. Similarly, the most commonly found plasmid-borne β-lactamase, *bla*_TEM-1_ (92%, 11/12), and the second most common, *bla*_CTX-M-14_ (75%, 9/12), were also ESBLs. These findings highlight the significant role of ESBLs in the development of antibiotic resistance in CRKP.

In addition to ESBLs, carbapenemases were also detected in 83% (10/12) of the isolates. The most commonly found carbapenemases was *bla*_KPC-2_ (58%, 7/12), followed by *bla*_OXA-48_ (2/12) and *bla*_IMP-8_ (1/12). Porin modification (GD insertion) or loss (Q305 stop codon) in OmpK36 was also found in 67% (8/12) of the strains, with no porin deficiency observed in OmpK35. Notably, all seven isolates that had acquired *bla*_KPC-2_ also had GD insertion in L3 of OmpK36. In the two carbapenemase-negative strains, OmpK36 was lost in one isolate (Pinhead-Larry) due to stop codon, while the other (Dirty-Dan) had no porin deficiency.

The copy number variations of β-lactamases were also identified in half of the CRKP isolates, with identical copies of the same β-lactamases successfully disambiguated through the use of long-read sequencing. Five β-lactamases (*bla*_OXA-48_, *bla*_OXA-10_, *bla*_TEM-1_, *bla*_CTX-M-14_, and *bla*_CMY-2_) were duplicated in six isolates. The presence of two copies of *bla*_OXA-48_, a well-known carbapenemase, on the chromosome was particularly noteworthy as it may further increase resistance to carbapenems. 

### 2.2. Investigation of Novel Stop Codon Associated with Carbapenemase-Negative CRKP

**Figure 2:**
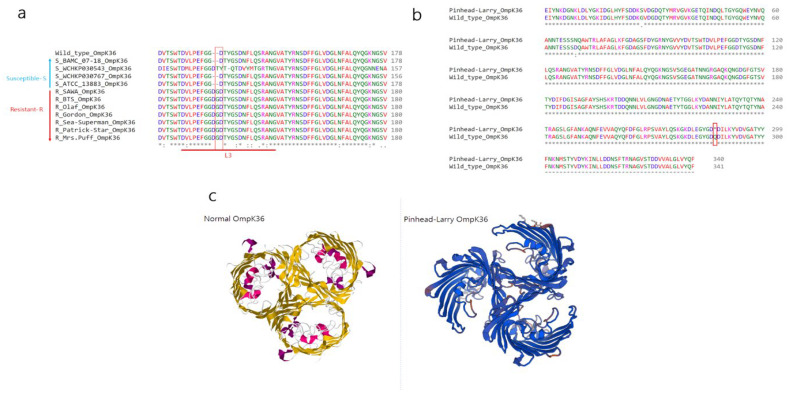
Characterization of a novel stop codon associated with carbapenemase-negative CRKP. The sequence alignment and structure reconstruction of OmpK36 in carbapenemase-negative and carbapenem-resistant K. pneumoniae isolates. (**a**) Multiple sequence alignment of the seven carbapenem-resistant isolates against five public carbapenem-sensitive strains of OmpK36 reveals GD insertion in loop L3. (**b**) Sequence alignment of OmpK36 between the isolate Pinhead-Larry and the K. pneumoniae reference strain reveals a Q309 stop codon. (**c**) Comparison of the protein structures between the wild type and Q309 stop codon OmpK36, providing a visual representation of the structural impact of the stop codon on the protein.

We identified two types of OmpK36 mutations in eight out of twelve isolates. Through sequence alignment against publicly available carbapenem-sensitive *K. pneumoniae* strains, we identified a known di-amino acid insertion (Gly-Asp; GD) in loop 3 of OmpK36. This insertion was found to restrict the diffusion of carbapenems (Figure 2a) [13]. Notably, the seven CRKP isolates with GD insertion in OmpK36 also acquired carbapenemase *bla*_KPC_, suggesting strong resistance to carbapenem. 

In addition, we identified a novel stop codon (Q309) at OmpK36 in a carbapenemase-negative CRKP isolate (Pinhead-Larry), resulting in a large truncation of the original OmpK36 (~367 AA, Figure 2b). Through comparison of protein structures using SWISS-MODEL, we observed that this truncation resulted in an unstructured and non-functional porin, in contrast to the structures of OmpK36 in other strains (Figure 2c). Therefore, we suggest that the loss of this porin, in conjunction with other beta-lactamases (*bla*_CMY_, *bla*_DHA_, and *bla*_TEM-1_), may contribute to carbapenem resistance even in the absence of carbapanemase.

### 2.3. Plasmidome Analysis Reveals the Importance of IncFII/IncR and IncC in CRKP

**Figure 3:**
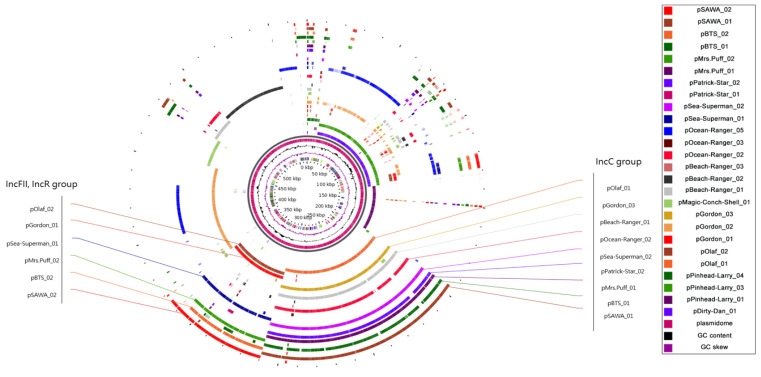
Plasmidome analysis of 26 complete plasmids from *K pneumoniae* genomes in the study. Plasmidome was constructed by concatenating the core genes of all plasmids. The core genes in each plasmid are depicted in different colors to indicate their individual presence. Two plasmid groups, IncF22/IncR and IncC, sharing common core genes are highlighted.

In order to further understand the distribution of β-lactamase resistance determinants among our study population, we performed a plasmidome analysis on the 26 complete plasmids from isolates in the study. We constructed a plasmidome by concatenating non-redundant genes in the 26 plasmids into a single reference and mapped all plasmids onto the reference (Figure 3, see Methods). The circular plasmidome revealed two major groups: IncFII/IncR and IncC (Supplementary Tables S4 and S5). Notably, plasmids within the same group exhibited conservation of encapsulated genes, suggesting that these plasmid groups may serve as conservative carriers of beta-lactamase resistance determinants.

### 2.4. Plasmid-Mediated Dissemination of β-Lactamase Resistance Determinants in CRKP: A Closer Look at IncFII/IncR and IncC Plasmid Groups

**Figure 4:**
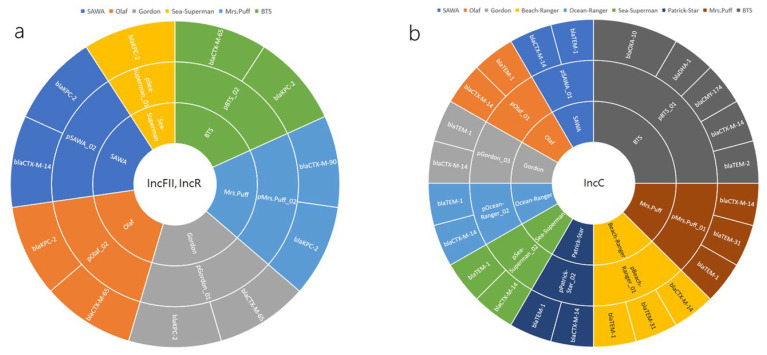
The correlation between plasmid groups and associated β-lactamases identified in the CRKP. The figure shows the frequency of different β-lactamase genes in relation to the plasmid groups IncFII/IncR (**a**) and IncC (**b**) groups. *bla*_KPC-2_ carriage is enriched with plasmids belonging to IncFII /Inc R group. *bla*_TEM_ carriage is frequently found on plasmids belonging to IncC group, while *bla*_CTX-M_ appears in both plasmid groups.

Our plasmidome analysis revealed a strong correlation between specific plasmid groups and associated β-lactamase resistance determinants in CRKP isolates. Specifically, our data suggest that the IncFII/IncR plasmid group is closely associated with the presence of the *bla*_KPC-2_ gene. All six IncFII/IncR plasmids in our study population encoded *bla*_KPC-2_, and five of them also contained *bla*_CTX-M_ (14, 65, 90) (Figure 4a). On the other hand, the IncC plasmid group was found to be closely associated with the presence of the *bla*_TEM-1_ and *bla*_CTX-M-14_ genes. All nine IncC plasmids in our study population contained these genes (Figure 4b). These findings suggest that these plasmid groups may have played a significant role in the circulation and maintenance of certain β-lactamase resistance determinants among CRKP isolates.

### 2.5. Global Phylogenetic Analysis of Plasmids in CRKP: Evidence of Endemic IncC in Taiwan

**Figure 5:**
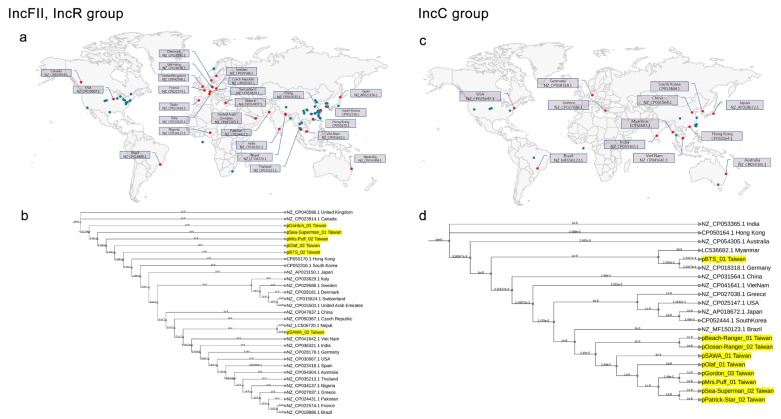
Global phylogeny of plasmids in CRKP reveals endemic IncC in Taiwan. (**a**) Global distribution of IncFII and IncR plasmids reveals a diverse distribution pattern among different regions. (**b**) Phylogenetic analysis of IncFII and IncR plasmids demonstrates the high diversity and evolutionary dynamics of these plasmids. (**c**) Global distribution of IncC plasmids shows that this plasmid group is prevalent in Taiwan, suggesting the presence of endemic transmission of IncC plasmids in this region. (**d**) Phylogenetic analysis of IncC plasmids reveals local clusters of related plasmids, further supporting the possibility of sustained local transmission of IncC plasmids in Taiwan.

To further understand the potential mechanisms of the spread and transmission of carbapenem resistance among *K. pneumoniae*, we performed a global phylogenetic analysis of the plasmids in our study population (Figure 5). Our analysis revealed a distinct distribution of the IncFII/IncR plasmids, with a diverse range of clades observed globally (Figure 5a,b). Interestingly, we found a high prevalence of the IncC plasmids in Taiwan, potentially indicating sustained local transmission of these plasmids (Figure 5c,d). Our phylogenetic analysis also demonstrated the diversity of the IncC plasmids, with several local clusters observed. These findings suggest that the IncC plasmids may play a significant role in the spread and transmission of carbapenem resistance among *K. pneumoniae* populations in Taiwan.

### 2.6. The Evolution and Expansion of IS26 Integrons: A Driving Force in Carbapenem Resistance

**Figure 6:**
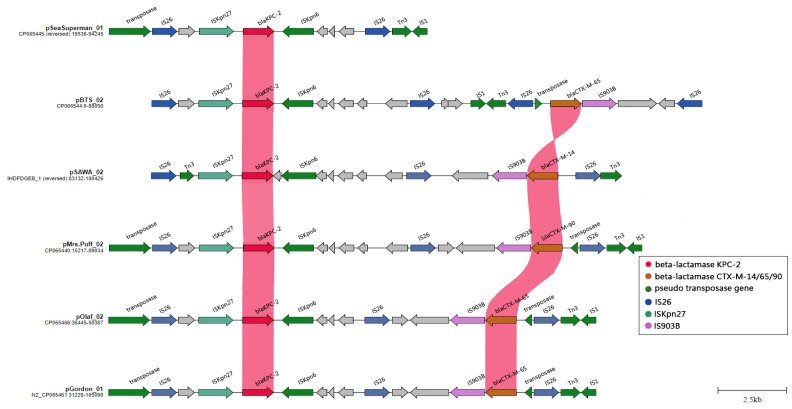
Tracing the evolution of IS26 integrons in plasmid IncFII from CRKP. The figure shows the novel structural organization of IS26-*bla*_KPC-2_-*bla*_CTX-M_ elements in plasmid IncFII. The progression from the common form 1 (pSeaSuperman_01), to the intermediate form 2 (pBTS_02), and finally to the advanced form 3 (lower 4 plasmids) suggests that IS26 integrons have undergone evolution and expansion, which may have contributed to the development of carbapenem resistance in these *K. pneumoniae* strains.

In our study, we aim to investigate the evolution and expansion of IS26 integrons in a sample of CRKP isolates. Utilizing long-read sequencing, we were able to uncover a novel structural organization of IS26-*bla*_KPC-2_-*bla*_CTX-M_ elements within plasmid IncFII. Our analysis identified an expansion from the common form IS26-*ISKpn27*-*bla*_KPC_-*ISKpn6*-IS26 (pSeaSuperman_01), to an intermediate form IS26-*ISKpn27*-*bla*_KPC_-*ISKpn6*-IS26-IS26-*bla*_CTX-M_-IS903B-IS26 (pBTS_02), and ultimately to form IS26-*ISKpn27*-*bla*_KPC_-*ISKpn6*-IS26-IS903B-*bla*_CTX-M_-IS26, which was observed in four of the plasmids (Figure 6). Moreover, the plasmid-encoding *bla*_OXA-48_ is located within the following integron structure: IS4-IS1-LysR-*bla*_OXA-48_-IS1-IS4. Similarly, the two chromosome-encoding *bla*_OXA-48_s are also surrounded by IS4 and IS1.

## 3. Discussion

In the current study, we investigated the molecular mechanisms and evolutionary dynamics of carbapenem resistance in *K. pneumoniae*. Through the third-generation platform-based complete genome sequence, we were able to identify the presence of complex β-lactamase genes, including ESBL, AmpC, and carbapenemase, along with diverse OmpK 36 mutations, including a novel Q309 stop codon in our study strains. Our phylogenetic analysis revealed the circulation of dominant plasmid types, including IncFII/IncR and IncC, among the CRKP strains. Additionally, we discovered the presence of IS26 integrons in these plasmids, which were found to have undergone a process of evolution and expansion. Our results suggest that the spread of these plasmids and integrons may have contributed to the development and spread of carbapenem resistance in *K. pneumoniae*.

One of the major findings in our study is the identification and characterization of the IS26 integrons in plasmid IncFII/IncR from CRKP, which revealed a progression from the common form 1 (pSeaSuperman_01), to an intermediate form 2 (pBTS_02), and ultimately to the advanced form 3 (Figure 6). This progression suggests that the IS26 integrons in these plasmids have undergone a process of evolution and expansion. An in-depth analysis of the surrounding structure of *bla*_KPC-2_ revealed that *bla*_KPC-2_ is located in a gene fragment with IS26 repeats at both ends and with *ISKpn27* upstream and *ISKpn6* downstream in the middle (pSeaSuperman_01). Similar genetic structures of *bla*_KPC-2_ were found in pBTS_02, with a combination of IS26-IS *Kpn27*-*bla_KPC−2_*-IS *Kpn6*-IS26 and IS26-*bla*_CTX-M_-IS903B-IS26. IS26-IS *Kpn27*-*bla_KPC−2_*-IS *Kpn6*-IS26 and *bla*_CTX-M_ flanked by IS26 and IS903B were observed in pSAWA_02, pMrs.Puff_02, pOlaf_02, and pGordon_01. The dissemination of *bla*_KPC-2_ has been reported by the horizontal transfer of IS*26*-IS *Kpn27*-*bla_KPC−2_*-IS *Kpn6*-IS *26* unit [14]. This underscores the capability of IS26 integrons to acquire and spread antibiotic-resistant genes, thereby contributing to the growth of multidrug resistance and playing a significant role in the dissemination of CRKP.

The high prevalence of plasmid-mediated resistance determinants in CRKP isolates, specifically the presence of plasmid groups IncFII/IncR and IncC which exhibit conservation of encapsulated genes, may serve as conservative carriers of beta-lactamase resistance determinants. The correlation between plasmid groups and the associated beta-lactamases identified in the CRKP indicate that *bla*_KPC-2_ carriage is enriched with IncFII and IncR, *bla*_TEM_ carriage is frequently found on IncC, and *bla*_CTX-M_ appears in all groups. IncFII/IncR plasmids are typically characterized by their low copy number and narrow host range and are primarily found in Enterobacterales. Although the presence of KPC-2 encoding IncFII/IncR plasmids has been reported in China [15], our global phylogeny study of the plasmids in the CRKP reveals endemic IncC in Taiwan, suggesting local transmission and the need for control strategies in that region.

In recent years, there has been growing evidence to suggest that IncC plasmids play a significant role in the dissemination of carbapenem resistance in Asia. These plasmids, known for their broad host range and ability to capture various resistance genes, have been increasingly reported in carbapenem-resistant bacterial isolates across the region [16]. IncC plasmids often carry carbapenemase genes, which confer resistance to carbapenem antibiotics [17]. In addition, the presence of IncC plasmids has been linked to the spread of multidrug resistance among various Gram-negative bacterial species [18]. The association between IncC plasmids and carbapenem resistance in Asia highlights the importance of continuous surveillance and monitoring of these mobile genetic elements, as well as the development of targeted interventions to limit their spread and mitigate the impact of antibiotic resistance in the region.

In our study, third-generation whole-genome sequencing reveals high complexity of β-lactamase genes copy number variation. We identified copy number variation in five β-lactamase genes encoding ESBL, AmpC, and carbapenemase, which represent significant enrichment of these families. The five different β-lactamase genes with copy number variation are found across chromosomes and plasmids. Moreover, the copy number variation in CRKP is widespread, as 50% of tested strains carry at least one of them. Our results demonstrate that the β-lactamase genes copy number variation is not uncommon in CRKP, emphasizing the need to identify the contribution of each copy number variation to antimicrobial resistance and to track their spread as the use of broad-spectrum antimicrobial agents intensifies and as the development of therapeutic strategy targeting β-lactamase continues. Polymorphisms in genetic copy numbers are associated with gene expression and coding sequence, making them important factors in the evolutionary process. Increasing copy number of ESBL, AmpC, and a variety of β-lactamase genes have been reported in carbapenem-resistant Enterobacterales [19,20,21,22]. Consistent with previous findings, amplification of β-lactamase encoding genes and porin disruption is common in CRKP, regardless of the presence of carbapenemase [23]. We further demonstrated carbapenemase and ESBL genes co-amplification in CRKP (strain Ocean-Ranger). The appreciation for the complexity of β-lactamase gene amplification in CRKP emphasizes the need to develop new surveillance systems to better define its scope and clinical impact.

A key finding was the identification of a novel stop codon (Q309) at OmpK36 in non-CP-CRKP, leading to a truncated OmpK36. Chromosome-based modifications of the major porins of the outer membrane, OmpK36, limit antibiotic influx across the outer membrane in CRKP. These changes combined with ESBL result in a decrease in carbapenem concentrations at the site of their transpeptidase targets, the periplasm, abrogating their bactericidal action [2]. By comparative genomics and solving the crystal structure of clinical non-CP-CRKP OmpK36 variants, we provide direct structural evidence of large truncation, mediated by a novel stop codon (Q309), resulting in OmpK36 porin loss. In the presence of AmpC amplification and ESBL, the findings illustrate the complex genetics underlying CRKP. Our results suggest that the ongoing selective pressure imposed by the extensive use of carbapenems in hospitals drives the growth of KP-expressing Q309 stop codon mutants. This has further expanded our understanding of OmpK36 porin mutation associated with CRKP.

Previous studies have reported the detection of carbapenem-resistant strains, including those harboring OmpK36 mutation, in various sources [13]. According to a study in 2022, mutations in outer membrane porins play an important role in mediating resistance to carbapenems, a key class of antibiotics. In particular, mutations that mediate pore constriction have been shown to consist of amino acid insertions in extracellular loop 3 (L3) of OmpK36, a motif that conformationally determines the minimal pore radius. Strains harboring L3 insertions remain susceptible to novel drugs, including beta-lactam/beta-lactamase inhibitor combinations. This study highlights the importance of monitoring the emergence and spread of strains with OmpK36 L3 insertions for the control of resistant KP infections and provides crucial data for drug development and treatment strategies [13]. Although our study focuses on the endemic occurrence of CRKP in Taiwan, these findings highlight the importance of considering the broader environmental context and the potential for the dissemination of resistant strains across different ecosystems.

Although our study does not directly investigate the comparative fitness or virulence of the endemic strains in Taiwan, some factors may contribute to their potential for increased robustness or risk to human health. The presence of specific resistance genes, plasmids (such as IncC), and outer membrane protein mutations (e.g., in OmpK36) may enhance the survival and adaptability of these strains in the presence of antibiotics, facilitating their persistence and transmission in various environments [2,24]. Additionally, certain virulence factors, such as hypermucoviscosity, siderophore production, and capsule synthesis, have been associated with increased pathogenicity in some CRKP strains [25,26]. Further research is needed to determine whether the endemic strains circulating in Taiwan possess these or other virulence factors and to evaluate their potential impact on human health.

The increasing prevalence of carbapenem-resistant strains, including those endemic to specific regions, such as Taiwan, highlights the urgent need for effective control strategies and monitoring efforts to limit the spread of antibiotic resistance. Comprehensive control strategies and monitoring efforts tailored to both local and international contexts should encompass antibiotic stewardship programs that promote the appropriate use of antibiotics in healthcare settings and the community, as well as regular monitoring of antibiotic resistance patterns and the prevalence of specific resistance determinants, such as carbapenemases, OmpK36 mutations, and IncC plasmids [27,28]. Environmental controls are also crucial, focusing on reducing contamination of water, soil, and food sources through improved waste management, regulation of antibiotic use in agriculture, and monitoring of antimicrobial residues in the environment [29]. Recognizing that antibiotic resistance is a complex problem involving human, animal, and environmental health, a One Health approach that integrates strategies across these domains is essential for effective control, promoting intersectoral collaboration, research, and policy development to address the multifaceted drivers of resistance [30]. By implementing these control strategies and monitoring efforts in a coordinated manner, we can better respond to the growing challenge of carbapenem resistance, both in the context of endemic strains and the broader global landscape.

This study provides insight into the mechanisms of resistance in CRKP, highlighting the prevalence and diversity of carbapenem-resistant determinants and porin modifications in the study population. Notably, we identified copy number variation of β-lactamases in half of the carbapenem-resistant *K. pneumoniae* isolates. Identical copies of the same β-lactamases were successfully disambiguated due to long-read sequencing. Five β-lactamases, namely, *bla*_OXA-48_, *bla*_OXA-10_, *bla*_TEM-1_, *bla*_CTX-M-14_, and *bla*_CMY-2_, were duplicated in six isolates. The two copies of *bla*_OXA-48_, a well-known carbapenemase, were encoded on the chromosome, while the others were plasmid-borne. *bla*_OXA-10_ can weakly hydrolyze carbapenems [31]. Hence, duplication of these β-lactamases may further increase their resistance to carbapenems.

## 4. Materials and Methods

### 4.1. Bacterial Isolates and Antibiotic Susceptibility Testing

In this study, 12 blood isolates of carbapenem-resistant *Klebsiella pneumoniae* bacteremia were collected (Supplementary Table S1). Species identification was performed using Matrix-assisted laser desorption/ionization time-of-flight mass spectrometry (MALDI-TOF/MS) (bioMérieux, Marcy-l’Étoile, France). Antimicrobial susceptibility testing for cefazolin, gentamicin, amikacin, trimethoprim-sulfamethoxazole, imipenem, ceftriaxone, ceftazidime, and cefepime was conducted using the VITEK^®^2 system (bioMérieux Inc., Durham, NC, USA) according to the Clinical and Laboratory Standards Institute (CLSI) breakpoints. The minimum inhibitory concentration (MIC) of colistin was determined by broth microdilution and susceptibility was interpreted according to the European Committee on Antimicrobial Susceptibility Testing (EUCAST) guidelines (susceptible, MIC ≤ 2 mg/L; resistant, MIC > 2 mg/L).

### 4.2. DNA Extraction, Whole-Genome Sequencing, Assembly, and Prediction of Antimicrobial Resistance Determinants

DNA was extracted from each CRKP isolate using the Qiagen DNeasy blood and tissue kit (Qiagen Co., Hilden, Germany). Nanopore DNA library construction was performed using the Rapid Barcoding Kit, and sequencing was conducted in an R9.4 flow cell with the GridIon device (Oxford Nanopore Technologies). The basecalled reads were assembled into chromosomes and plasmids using the Flye assembler (https://github.com/fenderglass/Flye) (accessed on 2 March 2021) [32], and then polished using Racon (https://github.com/lbcb-sci/racon) (accessed on 2 March 2021) [33], Medaka (https://github.com/nanoporetech/medaka), (accessed on 2 March 2021) and Homopolish (https://github.com/ythuang0522/homopolish) [34] (accessed on 2 March 2021). The protein-coding genes were annotated by the NCBI Prokaryotic Genome Annotation Pipeline (PGAP). The resistome in each polished genome was annotated by both NCBI AMRFinderPlus [35] and the Comprehensive Antibiotic Resistance Database [36]. MLST of Klebsiella pneumoniae was carried out on PubMLST [37].

### 4.3. Analysis of Plasmidome

The protein-coding genes in all plasmids were extracted, duplicated copies were removed, and a reference was constructed by concatenating all non-redundant gene sequences. Each plasmid was aligned onto the reference using BLAST and visualized using GView (https://github.com/phac-nml/gview-wiki/wiki, accessed on 2 March 2021). Each plasmid was confirmed and classified by PlasmidFinder [38]. The sequence has been deposited in GenBank under the accession number CP065436-CP065472.

## 5. Conclusions

The current carbapenemase-centered approach in both surveillance and diagnostics for CRKP may not be sufficient to control CRKP spread and treat CRKP infections. Further development of control measures and therapeutic regimens targeting resistance evolution are warranted [39]. This information provides valuable insights into the mechanisms of antibiotic resistance and the spread of plasmids among bacterial populations. By identifying these plasmid groups as conservative carriers of beta-lactamase resistance determinants, we can better understand the distribution and spread of antibiotic resistance in *K. pneumoniae*, and develop targeted strategies to combat the spread of antibiotic resistance in this important pathogen.

Our findings indicate that the IS26 integrons have acquired and disseminated *bla*_KPC-2_ and *bla*_CTX-M_ genes, which are responsible for carbapenem resistance. Furthermore, the progression suggests that these integrons have undergone a process of evolution and expansion, which may have contributed to the development of carbapenem resistance in these *K. pneumoniae* strains. This highlights the importance of monitoring the evolution and spread of IS26 integrons and the need for strategies to control their spread in order to prevent the emergence of multidrug-resistant bacteria.

## Data Availability

The assembled genome and gene annotations have been deposited at NCBI/GenBank under the accession numbers CP065436—CP065472.

## Supplementary Materials

The following are available online at https://www.mdpi.com/article/10.3390/antibiotics12040749/s1, Table S1. Antimicrobial susceptibility tests profile of K. pneumoniae in the study; Table S2. Summary of genome assembly data; Table S3. Summary of identified plasmids and plasmid-encoded β-Lactamase genes; Table S4. Summary of assigned Incompatibility group C (IncC) plasmids in the study; Table S5. Summary of assigned Incompatibility group C (IncFII) plasmids in the study.
